# Serum YKL-40 Levels and Chitotriosidase Activity in Patients with Beta-Thalassemia Major

**DOI:** 10.1155/2014/965971

**Published:** 2014-04-08

**Authors:** Maria Musumeci, Vincenzo Caruso, Emilia Medulla, Venerando Torrisi, Roberta Migale, Silvia Angeletti, Salvatore Musumeci

**Affiliations:** ^1^Center for Integrated Research, Department of Laboratory Medicine and Microbiology, Campus Bio-Medico University of Rome, 00128 Rome, Italy; ^2^Center of Microcitemia, 95123 Catania, Italy; ^3^IRMA, Acireale, 95024 Catania, Italy; ^4^Department of Surgery and Cancer, Parturition Research Group, Institute of Reproduction and Developmental Biology, Imperial College London, London W12 0NN, UK; ^5^Department of Chemical Sciences, University of Catania and Institute of Biomolecular Chemistry, CNR, 95125 Catania, Italy

## Abstract

*Background*. YKL-40 association with human disease has been the object of many years of investigation. **β**-thalassemia patients are affected by hepatic siderosis, which determines a fibrotic process and tissue remodelling. Chitotriosidase has been found to be increased in thalassemic patients returning to normal in patients submitted to bone marrow transplantation. YKL-40 is associated with macrophage activation in liver and in other tissues. The aim of the study was to analyse the level of serum YKL-40 and plasma chitotriosidase activity of patients with beta-thalassemia to assess whether their expression correlates with liver disease and degree of liver siderosis. *Methods*. Expression of YKL-40 and chitotriosidase as a marker of inflammation in 69 thalassemic patients were evaluated. We sought to investigate whether these two chitinases could be considered as a significant biomarker to evaluate therapy effectiveness. *Results*. Surprisingly we found normal value of YKL-40. We, also, analysed chitotriosidase activity in the same patients that was slightly increased as a consequence of macrophage activation. *Conclusions*. These data would suggest a good treatment for these patients.

## 1. Introduction

The glycosyl hydrolase family 18 of chitinases is an ancient gene family widely expressed from prokaryotes to eukaryotes. In mammals, despite the absence of endogenous chitin, a number of chitinases and chitinase-like proteins have been identified and their role is yet to be fully elucidated. YKL-40 is a highly conserved glycoprotein belonging to the family of glycosyl hydrolase 18. The YKL-40 (also termed chitinase 3-like 1) inhibits oxidant-induced lung injury, augments adaptive Th2 immunity, regulates apoptosis, stimulates alternative macrophage activation, and contributes to fibrosis and wound healing [1].

The YKL gene is localized on chromosome 1q32.1 and two different splice forms are reported: isoform 1 containing exon 1–10 and isoform 2 in which exon 8 has been spliced out [2]. YKL-40 is expressed by macrophage cells during late stage of differentiation [3], by tumor-associated macrophages [4], by infiltrating macrophages in various inflammatory conditions such as rheumatoid arthritis and osteoarthritis [5], and by macrophage and giant cells in arteritis vessel [6]. YKL-40 is also overexpressed in macrophages of early atherosclerotic lesions [7] and in macrophage of bronchial tissue [8].

Increased YKL-40 protein expression is noted in patients with alcoholic liver disease and concurrent chronic hepatitis C virus infection [9, 10]. YKL-40 is not expressed in normal liver tissue except in mesenchymal structure of portal tract [11]. Interestingly, in chronic viral hepatitis portal tracts are the primary sites of fibrotic tissue formation from which fibrotic liver disease will drive the changes in liver architecture associated with the infection.

Although a wide body of evidences associates YKL-40 expression with several pathologies, its biological role is still poorly understood.

*In vitro *studies suggested a role for YKL-40 as a proliferation factor in synovial cells and chondrocytes and a suppressive effect on cytokine signalling in connective tissue cells [12, 13].

Chitotriosidase (Chit) is a chitinolytic enzyme selectively produced by activated macrophages. Serum chit activity is significantly higher in individuals suffering from atherosclerotic diseases, Gaucher disease (beta-glucocerebrosidase deficiency) [14], sarcoidosis [15], malaria [16], and thalassemia [17].

Beta-thalassemia is a haematological disorder caused by a genetic defect in the synthesis of beta globin chains, resulting in severe haemolytic anaemia, inefficient erythropoiesis, and enormous expansion of reticuloendothelial system [18]. Life-long transfusion regimens are essential to alleviate anaemia. Although regular blood transfusions have largely improved the prognosis, iron overload, especially in the liver (liver siderosis), and high risk of hepatitis C infections constitute a real threat to the quality of life of beta-thalassemia patients [19]. In beta-thalassemia patients both iron overload and HCV-related liver disease lead, albeit through different mechanisms, to hepatocellular necrosis, fibrosis, and cirrhosis. An iron chelation treatment is necessary to counteract the damaging effects of siderosis. Controlled regimens of blood transfusions together with interferon treatment of chronic hepatitis C have significantly improved the life of patients in duration and quality [20].

Plasma chitotriosidase activity was found to be increased to a variable extent in Sicilian patients diagnosed with beta-thalassemia major [17, 21]. All patients showed peripheral anaemia and considerable enlargement of the reticuloendothelial system. Since YKL-40 is associated with macrophage activation in liver and in other tissues, the aim of this study was to analyse the levels of YKL-40 and chitotriosidase activity in plasma of patients with beta-thalassemia to assess whether their expression correlates with liver disease and degree of liver siderosis. Additionally we sought to investigate whether these two chitinases could be considered as a significant biomarker to evaluate therapy effectiveness.

## 2. Material and Methods 

### 2.1. Patients

69 Sicilian patients with beta-thalassemia, 34 males and 35 females, were enrolled in this study (median age 33, range 15–69). The blood transfusion regimen started since the 24 months of age, associated to iron chelating therapy (40 mg/kg/day) according to* ad hoc* Italian protocol. Liver biopsy was performed in 20 patients, when serum transaminases resulted constantly elevated with viral hepatitis markers. A group of 21 healthy subjects (median age 38, range 27–47 years) were enrolled as controls. They were characterized by not being on medication and having no sign of preexisting disorders such as joint, liver, metabolic or endocrine diseases, or malignancy.

### 2.2. Clinical and Serological Parameters

Blood samples were collected before the last transfusion of concentrated red cells, between 8 and 12 a.m. Serum and plasma samples were analyzed either immediately or snap-frozen and stored at −80°C. Transaminases (ALT, AST), gamma-GT (GGT), LDH, total proteins, albumin/globulin ratio, and ferritin levels were determined by routine methods. The sideruria was monitored in these patients after Desferal load (500 mg/day). Level of liver siderosis was measured with SQUID magnetometer and magnetic resonance technology.

### 2.3. Chitotriosidase Activity

The chitotriosidase assay was based on the method described by Hollak et al. [14]. Chitotriosidase activity was measured by incubating 5 *μ*L plasma with 100 *μ*L of 22 *μ*mol/L 4-methylumbelliferyl-b-d-N,N8,N9 triacetylchitotriose (Sigma Chemical CO) in citrate-phosphate buffer, pH 5.2, for 15 min at 37°C. The reaction was stopped by the addition of 2 mL of 0.5 mol/L Na_2_CO_3_-NaHCO_3_ buffer, pH 10.7, and the fluorescence was read on a Perkin Elmer fluorimeter with excitation of 365 nm and emission of 450 nm. Chitotriosidase activity was measured as nanomoles of substrate hydrolysed per minute per mL (nmol/mL/hr). Plasma chitotriosidase activity was measured in duplicate and coefficient of variation was less than 5% in all measurements.

### 2.4. YKL-40 Determination

Serum YKL-40 concentrations were determined by a commercial enzyme-linked immunosorbent assay (Quidel, Santa Clara, CA, USA). The intra-assay and interassay variations were 3.6% and 5.3%, respectively. The sensitivity of the assay was 20 ng/mL. The samples were measured in duplicate.

### 2.5. Statistical Analysis

The statistical analysis was carried out with Origin Software. The results are expressed as median and range. Correlations between the different parameters were calculated by Spearman *P* test. *P* values <0.05 were considered significant.

## 3. Results

Table 1 shows the median level of serum YKL-40 and plasma chitotriosidase in thalassemic patients and in healthy controls. Two patients showed low values of chitotriosidase (1.03 and 2.4), which may result from genetic polymorphisms of CHIT gene affecting its expression levels. Median levels of transaminases AST and ALT, GGT, albumin/globulin ratio, LDH, total proteins, ferritin, SQUID, and MR are also shown in Table 1. One patient was HBsAg positive and 51 were anti-HBs positive following HBV vaccination. 48 patients were anti-HCV positive; however, only in 16 patients HCV RNA was detected. Two patients of this group underwent interferon/ribavirin treatment for permanent high transaminase levels. Two patients were HIV positive in treatment with antiretroviral therapy. Only one patient was affected by intermediate thalassaemia. 38 patients were splenectomized.

**Figure 1:**
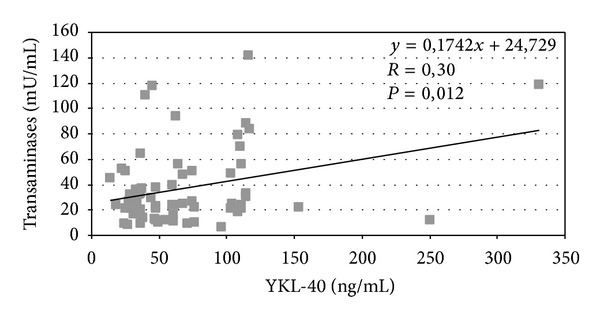
Correlation between serum transaminases and serum YKL-40 in 69 thalassemic patients.

**Figure 2:**
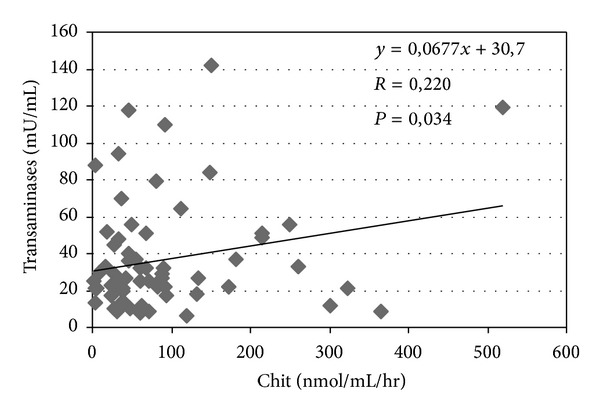
Correlation between serum transaminases and plasma chitotriosidase (Chit) in 69 thalassemic patients.

**Figure 3:**
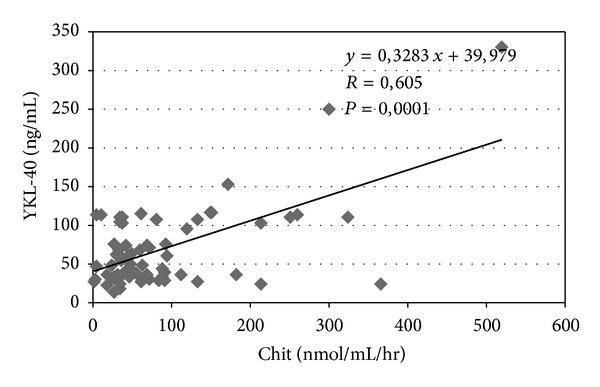
Correlation between serum YKL-40 and plasma chitotriosidase (Chit) in 69 thalassemic patients.

**Figure 4:**
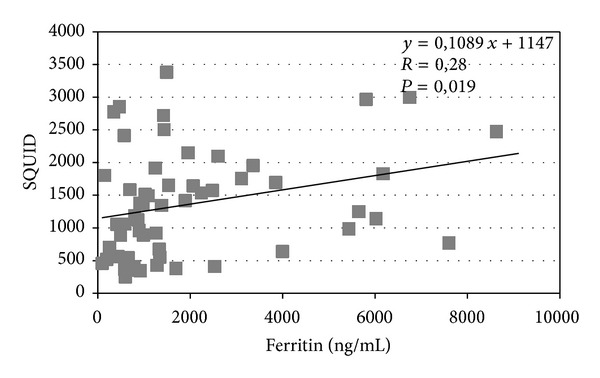
Correlation between SQUID and Ferritin in 69 thalassemic patients.

YKL-40 levels correlate with liver transaminases (*P* = 0.012) (Figure 1) and a similar correlation was observed between chitotriosidase and transaminases (*P* = 0.034) (Figure 2). The correlation between chitotriosidase and YKL-40 was significant (*P* = 0.0001) (Figure 3), supporting the hypothesis that the mechanisms regulating the expression of these two chitinases are interdependent. The correlation between SQUID and ferritin was significant (*P* = 0.019) (Figure 4). Correlation between YKL-40 and ferritin (*P* = 0.0008) and the correlation between chitotriosidase and ferritin were also significant (*P* = 0.00001). The liver biopsy showed different results with a prevalence of liver fibrosis and different degree of fibrosis (fibrosis 1° 4, fibrosis 2° 3, and mild fibrosis 2). Four patients showed chronic active hepatitis HCV+ with siderosis, while three showed siderosis and three chronic active hepatitis with fibrosis, five chronic low activity hepatitis (Lic 12, Lic 6.9, Lic 14.8, Lic 14, and Lic 15). Liver biopsy showed enlarged portobiliary space with fibrosis and lymphocytic infiltration, sclerosiderotic nodules, and signs of slight chronic hepatitis. No statistically significant correlation was found between histological alteration and level of YKL-40 and chitotriosidase.

## 4. Discussion

Due to a significant association of elevated concentration of plasma YKL-40 with various diseases, considerable effort has been devoted to the elucidation of its biological activity.

Increased serum concentrations of YKL-40 were observed in patients affected by pathologies involving extracellular matrix degradation and angiogenesis, such as rheumatoid arthritis [22], hepatic fibrosis [23], and osteoarthritis [24], as well as cancer [1].

Increased concentrations of YKL-40 were detected not only in tissue-localised inflammation, but also systemically in serum of patients with rheumatoid arthritis [25]. YKL-40 mRNA expression was higher in macrophages in early atherosclerotic lesions and in infiltrating macrophages [26]. YKL-40 can act as an adhesion and migration factor in vascular cells and it may have a role in molecular processes involved in vascular occlusion and heart development [27].

Increased production of YKL-40 is an indicator of liver pathology. YKL-40 is differentially upregulated in cirrhotic liver on the end-stage of hepatitis C virus- (HCV-) induced liver cirrhosis [28]. Serum level of YKL-40 correlates with its mRNA expression in liver [29] and was suggested to be a useful noninvasive marker for evaluation of the degree of fibrosis as well as therapy effectiveness in patients with HCV-associated liver disorders [30]. Increased plasma levels of YKL-40 were also suggested to reflect the progression of liver fibrosis in alcoholics [31].

Beta-thalassemia patients are affected by liver siderosis which determines a fibrotic process and an extracellular tissue remodelling. Given the strong association of YKL-40 with inflammation, higher levels of this molecule were expected.

However, in this study the level of YKL-40 was found to be lying in the normal range and only a slight correlation was found with the liver status as demonstrated by liver transaminases. Also the presence of anti-HCV antibodies in these patients, which is often associated with chronic hepatitis, does not correlate with YKL-40 levels. In other studies the elevated serum YKL-40 is associated with poor prognosis and survival in patients with alcoholic liver disease [32] and in patients with coronary artery disease [33]. A possible explanation of our results is maybe the timely and correct treatment of the thalassemia patients enrolled in this study. Indeed the chronic hepatitis was controlled by interferon/ribavirin therapy and no relapse was reported. Also, the compliance to iron chelating treatment was effective to reduce the liver siderosis. Moreover, a correct blood transfusion regimen is effective in reducing intestinal iron absorption and the hyperhemolysis, which are the main causative factors of liver siderosis.

We think that YKL-40 may be considered as a marker of correct treatment and its dosage could represent a new marker for prognosis. This last hypothesis could be supported by the observation that the elevated level of another chitinase, chitotriosidase, produced by activated macrophage, returns to normal values in patients following bone marrow transplant, which at the moment is considered as the best therapeutic option available [34]. Nevertheless, the levels of chitotriosidase in thalassemic patients are just slightly elevated compared to healthy individuals. These data suggest that in patients with thalassemia the improved treatment with regular transfusion regimen and the associated iron chelation therapy could result in an apparent clinical and laboratory recovery.

## 5. Conclusions

Our results could support the idea that current regimens of transfusions and the associated chelation therapy have definitely contributed to ameliorate the health status of thalassemia patients. This hypothesis could be, probably, explained by testing the chitinases levels in less developed countries where transfusion regimens and chelation therapy are not readily available.

## Figures and Tables

**Table 1:** Clinical and serological parameter in 69 thalassemic patients and healthy controls.

Parameters	Thalassemic patients	Reference values	Healthy controls	Spearman *P* test
Age (years)	33	—	38	*P* = 0.60
	(15–69)		(27–47)	
Sex M/F	34/35	—	10/11	—
AST U/L	23	Up to 37	17	*P* = 0.63
	(9–156)		(8–34)	
ALT U/L	26	Up to 78	27	*P* = 0.54
	(8–142)		(17–54)	
GGT U/L	21	15–85	20	*P* = 0.46
	(5–153)		(9–82)	
LDH U/L	325	0–248	170.5	*P* = 0.84
	(166–1082)		(123–208)	
Total proteins gr/dL	7.2	6.4–8.3	7.25	*P* = 0.07
	(6.5–7.25)		(6.5–8.25)	
Albumin/globulin ratio	1.56	1.10–2.40	1.55	*P* = 0.95
	(0.93–1.28)		(1.22–1.87)	
Ferritin ng/mL	1250	F 8–252	157	*P* = 0.52
	(89–9110)	M 26–388	(34–314)	
SQUID	1296	—	—	—
	(254–3379)			
MR	5.61	—	—	—
	(0.38–43)			
Chitotriosidase nmol/mL/hr	84.38	—	44	*P* = 0.005
	(1.03–519)		(0.6–73)	
YKL-40 *μ*g/L	37.6	—	29.18	*P* = 0.27
	(14–117.32)		(17.5–111.4)	
